# Automatic Segmentation and Measurement of Vasculature in Retinal Fundus Images Using Probabilistic Formulation

**DOI:** 10.1155/2013/260410

**Published:** 2013-12-08

**Authors:** Yi Yin, Mouloud Adel, Salah Bourennane

**Affiliations:** Institut Fresnel, Ecole Centrale de Marseille, Aix-Marseille Université, Domaine Universitaire de Saint-Jérôme, 13397 Marseille, France

## Abstract

The automatic analysis of retinal blood vessels plays an important role in the computer-aided diagnosis. In this paper, we introduce a probabilistic tracking-based method for automatic vessel segmentation in retinal images. We take into account vessel edge detection on the whole retinal image and handle different vessel structures. During the tracking process, a Bayesian method with maximum *a posteriori* (MAP) as criterion is used to detect vessel edge points. Experimental evaluations of the tracking algorithm are performed on real retinal images from three publicly available databases: STARE (Hoover et al., 2000), DRIVE (Staal et al., 2004), and REVIEW (Al-Diri et al., 2008 and 2009). We got high accuracy in vessel segmentation, width measurements, and vessel structure identification. The sensitivity and specificity on STARE are 0.7248 and 0.9666, respectively. On DRIVE, the sensitivity is 0.6522 and the specificity is up to 0.9710.

## 1. Introduction

Automatic vessel segmentation in medical images is a very important task in many clinical investigations. In ophthalmology, the early diagnosis of several pathologies such as arterial hypertension, arteriosclerosis, diabetic retinopathy, cardiovascular disease, and stroke [1, 2] could be achieved by analyzing changes in blood vessel patterns such as tortuosity, bifurcation, and variation of vessel width on retinal images.

Early detection and characterization of retinal blood vessels are needed for a better and effective treatment of diseases. Hence, computer-aided detection and analysis of retinal images could help doctors, allowing them to use a quantitative tool for a better diagnosis, especially when analyzing a huge amount of retinal images in screening programs.

Many methods for blood vessel detection on retinal images have been reported in the literature [3–5]. These techniques can be roughly classified into pixel-based methods [6–14], model-based methods [15–21], and tracking-based approaches [22–29], respectively.

Pixel-based approaches consist in convolving the image with a spatial filter and then assigning each pixel to background or vessel region, according to the result of a second processing step such as thresholding or morphological operation. Chaudhuri et al. [8] used 2D Gaussian kernels with 12 orientations, retaining the maximum response. Hoover et al. [6] improved this technique by computing local features to assign regions to vessel or background. A multithreshold scheme was used by Jiang and Mojon [9], whereas Sofka and Stewart [10] presented a multiscale matched filter. Zana and Klein [11] used morphological filter combined with curvature evaluation for retinal segmentation. Neimeijer et al. [12] used a classification scheme based on a simple feature computation. Gabor wavelet transform with a Bayesian classification is performed by Soares et al. [13]. Staal et al. [7] applied a supervised classification based on features computed near the centerline. The same scheme was used by Ricci and Perfetti [14] but with a modified line operator to train a supervised pixel classifier.

Model-based approaches use parametric models to extract vessels. Al-Diri et al. [30] extracted blood vessel segment profiles and measured vessel width using a parametric active contour, based on two contours coupled by spring models. Active contour model was applied by Kozerke et al. [15] to automatically segment vessels. A level set geometric-based regularization approach is given by Gooya et al. [16]. Vasilevskiy and Siddiqi [17] developed FLUX for narrow elongated vessel segmentation and Law and Chung [18] improved this technique for spherical flux computation. Coronary arterial trees are reconstructed using elliptical parametric cross-section model by Kayikcioglu and Mitra [19]. Hessian-based method is proposed by Sato et al. [20] to characterize stenosis in coronary angiograms, whereas Wang et al. [21] used Hermite multiresolution model for retinal vessel analysis. A multiresolution approach based on a scale-space analysis is presented by Martínez-Pérez et al. [31], in which the width, the size, and orientation of retinal vessels are obtained.

Tracking-based approaches are based on local techniques, where either the centerline or vessel edges or both are extracted. Starting from seed points, these methods progress along vessels by iterative prediction and parameter estimation. Many methods including several 2D and 3D medical imaging modalities have been published [4]. An advantage of tracking methods is the guaranteed connectedness of vessel segment, whereas in pixels-processing-based methods, connectedness is not guaranteed. A whole vessel tree can be tracked by these methods without examining the vast majority of the image. Starting from the optic disc, Tolias and Panas [22] developed a fuzzy tracking model of 1D vessel profile. Their method, however, did not manage to handle branch points. Starting from seed points, Can et al. [23] used an iterative tracking algorithm, updating at each step the position and the orientation of vessel points. Zou et al. [24] developed a recursive tracking technique guided by accurate vessel direction and robust a priori knowledge and termination criteria. Compared to others, this approach could extract automatically most of the vessels with accurate vessel characteristics.

Among tracking methods, few probabilistic approaches have been reported in the literature [32–34]. The main ideas of a probabilistic tracking method have been presented in our previous work [35, 36], which needed some improvements like modeling blood vessels more accurately, handling different vessel configurations, and evaluating it on large database images.

In this paper, a novel fully automatic tracking based method using probabilistic formulation is introduced. First, seed points, located inside vessels, are obtained from the image. From these points, an iterative tracking algorithm detects simultaneously edges, diameter width, centerline, and orientation of the whole vascular tree. A branch prediction scheme to handle bifurcation and crossing is also developed. The major novelty lies in defining the likelihood of the hypothesis of edge points and the *a priori* probability based on Gibbs formulation. This new approach uses Gaussian model to approximate vessel sectional intensity profiles, identifies bifurcation and crossing structures using gradient analysis, tracks centerline and local vessel edges geometry, and improves the detection results as shown in the experiments and discussion section. A probabilistic segmentation scheme is associated with the maximum *a posteriori* (MAP) as a criterion to estimate local vessel edges.

In the following, Section 2 gives a general description of the proposed method.  Section 3 is devoted to the explanations of the tracking algorithm. Bayesian segmentation is described in Section 4. Finally, experiments on STARE, DRIVE, and REVIEW databases are presented and discussed in Section 5.

## 2. General Description of the Method

In this paper, we propose a tracking-based method to detect retinal vascular trees. First, a number of seed points are selected automatically on the retinal image, which provide initial parameters for the tracking algorithm. The tracking process starts then from each of the seed points and detects vessel edge points iteratively until end conditions are satisfied. Finally, when all the seed points are processed, the whole vascular tree is detected and the proposed algorithm stops.

A tracking process from one seed point is described in detail as follows.

### 2.1. Initialization

Initial parameters, which are obtained from a seed point, include initial vessel center point and vessel direction. The blood vessel is then detected by the tracking algorithm from the initial center point along the initial vessel direction.

### 2.2. Iteration

At a given step, a dynamic search window is defined based on local vessel parameters including vessel center point, direction, and diameter. We adopt a statistical sampling method to select new candidate edge points on the search window. A Bayesian method with the MAP as a criterion is used to pick out new vessel edge points from these candidate points. New vessel parameters are then updated for the next iteration. In addition, during the tracking process, a branch prediction scheme is applied at each iteration to detect vessel branches which exist in the local search area.

### 2.3. End

There are three end conditions for the proposed tracking process. Under each of these conditions, the tracking of current vessel stops. The three end conditions are as follows.

- Current vessel's end is considered to be found when the vessel's diameter is less than one pixel or the contrast between the vessel and background is low.
- Current vessel encounters a blood vessel which is already detected by a tracking process starting from another seed point.
- Vessel branches are found. All initial information of the branches is obtained by the algorithm and the tracking of these vessel branches starts. The branch with the biggest diameter is handled first.

## 3. Tracking Algorithm

### 3.1. Selection of Seed Points

In retinal image, blood vessels are not all connected because of the imaging conditions and different eye diseases. Therefore, the tracking algorithm starts from several initial points selected on the whole image and the tracking results are combined to get the final detected vascular tree. In this study, we use an automatic two-step method based on grid line analysis to pick out initial seed points. The first step, searching over grid lines, is similar to the procedure used by Can et al. [23]. The second step consists in filtering the image obtained in the previous step by the 2D Gaussian filter and getting the validated seed points.

**Figure 1:**
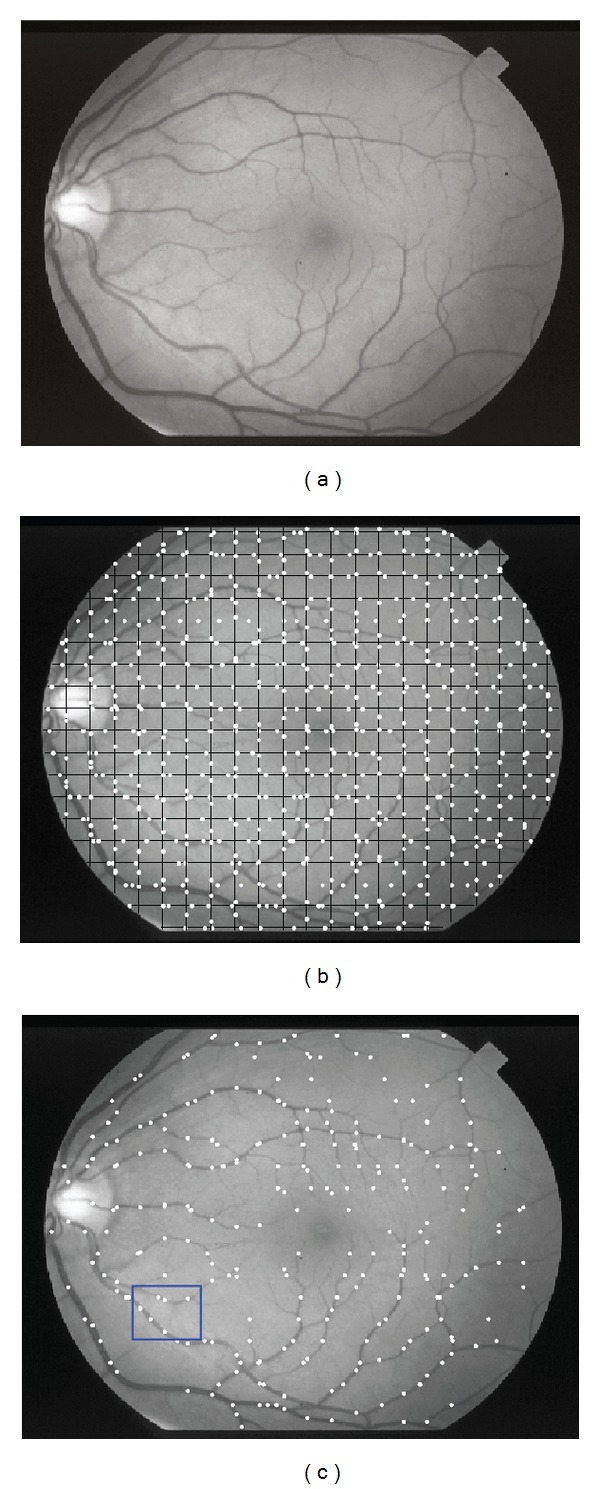
Selection of seed points on a retinal image: (a) retinal image; (b) grid lines and candidate seed points; (c) validated initial seed points.

First, a set of grid lines is drawn over the retinal image as shown in Figure 1(b). Local intensity minima on each grid line (horizontal or vertical) are detected (Figure 1(c)) and considered as candidate seed points. Among the detected local minima, some are related to noise or other eye tissues such as fovea. So, rejection of the false candidate seed points is needed in order to avoid unnecessary tracking. To test the validity of the candidates, we use a set of 2D Gaussian directional matched filters, which were first proposed by Chaudhuri et al. [8]. There are 12 different orientations for the Gaussian kernels which are spaced in 15° from each other. We convolve the 12 oriented filters with the given retinal image for the selected candidate points. If the highest response of 12 directional filters for a candidate seed point is above a local adaptive threshold, it is validated as a seed point and the corresponding filter direction is regarded parallel to local vessel direction. In our study, the local adaptive threshold is defined based on local intensity distribution. For a candidate seed point, the local adaptive threshold is computed as

(1) $$\begin{array}{c} T_{\text{seed}}=\mu_{\text{seed}}+\alpha \sigma_{\text{seed}}, \end{array}$$

where *μ*_seed_ and *σ*_seed_ are the mean grey level and standard deviation of the neighborhood of the candidate seed point, respectively. After many experiments, the value of parameter *α* is fixed to 1.2 and the size of the neighborhood is fixed to 61 × 61 pixels around each candidate seed point.

**Figure 2:**
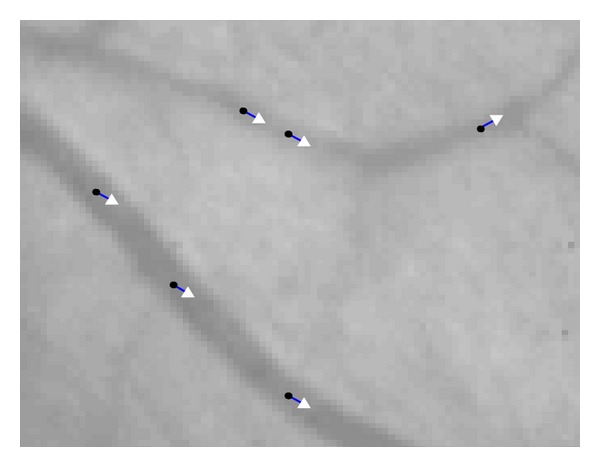
Examples of seed points.

This method is fast and effective because it considers only the pixels on the grid lines and validates seed points based on the 2D Gaussian filter. All the verified seed points are used for the initialization of the tracking algorithm. Each seed point is considered as local vessel center point and initiates the tracking algorithm twice, once in its related filter direction and the other along the opposite direction. If a seed point is out of vessels, the tracking algorithm stops after one or two iterations according to the end conditions. As shown in Figure 1(c), there is at least one seed point on each of the blood vessels. Some examples of seed points are shown in Figure 2, including locations and corresponding filter directions. Figure 2 is a subimage of Figure 1(c) marked by blue box.

### 3.2. Statistical Sampling

**Figure 3:**
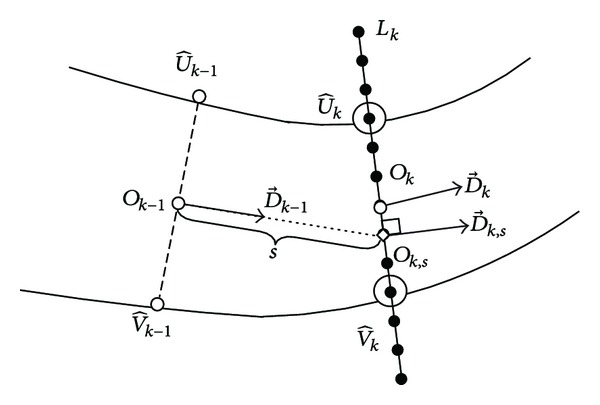
Linear search window: $\hat{U}$ and $\hat{V}$ are the vessel edge points, *O* is the center point, $\vec{D}$ is the vessel direction, and *k* is the index of the iteration. *L*_*k*_ is the linear search window at iteration *k*. Black points on *L*_*k*_ show the possible locations of new edge points.

The proposed tracking algorithm starts from each of the seed points and detects vessel edge points iteratively. During the tracking process, new vessel edge points are detected based on a statistical sampling scheme. As shown in Figure 3, at a given step, a line segment is obtained perpendicular to the tracking direction. This line segment is regarded as a linear search window, which restricts the possible locations of new vessel edge points. The width of the linear window is adaptive to local vessel diameter in order to cover the potential positions of new edge points.

At iteration *k* (when *k* = 0), the initial vessel center point *O*_0_ is an initial seed point. *O*_0_ may be not exactly in the middle of local vessel. However, this deviation will be corrected after two or three iterations. The initial vessel direction ${\vec{D}}_{}{}_{0}$ is along or opposite to the related filter direction of the initial seed point. A line segment is drawn centered on *O*_0_ and perpendicular to ${\vec{D}}_{}{}_{0}$, which is regarded as the initial linear window *L*_0_. The width of *L*_0_ is set bigger than the widest blood vessel in the retinal image. Two initial vessel edge points ${\hat{U}}_{0}$ and ${\hat{V}}_{0}$ are detected on *L*_0_ around the seed point by the proposed method. Initial vessel diameter is $d_{0}=|{\hat{U}}_{0}{\hat{V}}_{0}|$.

When *k* ≥ 1, vessel parameters at the previous iteration *k* − 1 are known, including vessel edge points ${\hat{U}}_{k-1}$, ${\hat{V}}_{k-1}$, center point *O*_*k*−1_, direction ${\vec{D}}_{}{}_{k-1}$ ($||{\vec{D}}_{}{}_{k-1}||=1$), and diameter *d*_*k*−1_. In order to get the linear window *L*_*k*_, we first extrapolate the vessel center point *O*_*k*−1_ to a point *O*_*k*,*s*_, which is *s* pixels away along ${\vec{D}}_{k-1}$. The extrapolated point *O*_*k*,*s*_ is described as

(2) $$\begin{array}{c} \vec{O_{k-1}O_{k,s}}=s{\vec{D}}_{k-1}. \end{array}$$

The vessel direction at *O*_*k*,*s*_ is obtained by performing gradient analysis of this point (see Section 3.3) and is denoted by ${\vec{D}}_{k,s}$. A line segment is then drawn centered on *O*_*k*,*s*_ and perpendicular to ${\vec{D}}_{k,s}$ as shown in Figure 3. This line segment is regarded as the linear search window *L*_*k*_ at iteration *k*. The width of the linear window is set twice the diameter of local blood vessel *d*_*k*−1_.

We select *N*_*L*_*k*__ points which are numbered from 1 to *N*_*L*_*k*__ on *L*_*k*_ (*k* ≥ 0). The interval between two adjacent points is fixed to 1 pixel. *N*_*L*_*k*__ selected points are considered as candidate edge points. New edge points ${\hat{U}}_{k}$ and ${\hat{V}}_{k}$ are chosen from these candidates by a Bayesian method (see Section 4). Then, new vessel parameters at iteration *k* are updated accordingly. The new center point *O*_*k*_ is the middle point of $\left[{\hat{U}}_{k},{\hat{V}}_{k}\right]$; vessel direction ${\vec{D}}_{k}$ is defined as the direction at point *O*_*k*_ by gradient analysis (see Section 3.3); and new vessel diameter is $d_{k}=|{\hat{U}}_{k}{\hat{V}}_{k}|$.

### 3.3. Gradient Analysis

In this study, the vessel direction at a point is assumed to be parallel to the nearest blood vessel. This direction can be estimated based on the local gradient information. Let the gradient vector at point (*i*, *j*) in the image has the polar representation. The modulus *G*_*ij*_ and the angle *θ*_*ij*_ are obtained based on the classic Sobel mask. Since the projection of a vector is maximized in the direction parallel to it, the dominant gradient direction at (*i*_*o*_, *j*_*o*_) is assumed as the optimum direction in which the projections of the gradient vectors in the neighborhood *M* × *N* around (*i*_*o*_, *j*_*o*_) are maximized. In practice, the size of neighborhood used for calculating the gradient direction is fixed to 5 × 5. If the angle of the dominant gradient direction at (*i*_*o*_, *j*_*o*_) is denoted by *θ*_*g*_, the projection function of the gradients is expressed as follows [41]:

(3) $$\begin{array}{c} F\left(\theta_{g}\right)=\underset{(i,j)\in M*N}{\sum }G_{ij}^{2}\cos^{2}\left(\theta_{ij}-\theta_{g}\right). \end{array}$$

*F*(*θ*_*g*_) is maximized by calculating the value of *θ*_*g*_ when making its derivative equal to zero. The angle of the dominant direction at (*i*_*o*_, *j*_*o*_) is

(4) $$\begin{array}{c} {\hat{\theta }}_{g}=\frac{1}{2}\arctan\left(\frac{\sum_{(i,j)\in M*N}^{}G_{ij}^{2}\sin\left(2\theta_{ij}\right)}{\sum_{(i,j)\in M*N}^{}G_{ij}^{2}\cos\left(2\theta_{ij}\right)}\right). \end{array}$$

On a grayscale image, the gradient vector points to the direction of the greatest change of the grey level. Therefore, the dominant gradient direction at point (*i*_*o*_, *j*_*o*_) is perpendicular to the nearest blood vessel on the retinal image. The angle of the vessel direction at (*i*_*o*_, *j*_*o*_) can be estimated as

(5) $$\begin{array}{c} \theta_{i_{o}j_{o}}={\hat{\theta }}_{g}\pm \frac{\pi }{2}. \end{array}$$

The sign before *π*/2 is determined by local vessel parameters as discussed later. Finally, the vessel direction at (*i*_*o*_, *j*_*o*_) is described as a unit vector by *θ*_*i*_*o*_*j*_*o*__:

(6) $$\begin{array}{c} \vec{D}{}_{i_{o}j_{o}}=\left(\cos\left(\theta_{i_{o}j_{o}}\right),\sin\left(\theta_{i_{o}j_{o}}\right)\right). \end{array}$$

**Figure 4:**
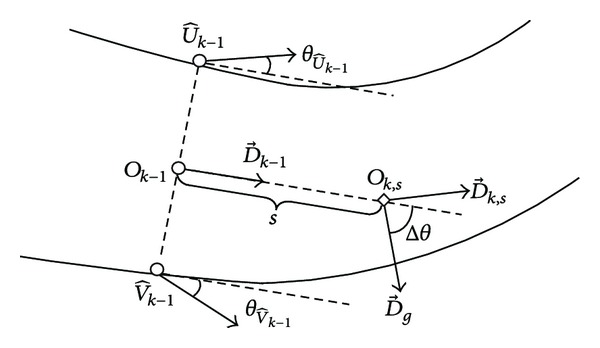
Calculation of the vessel direction at the extrapolated point *O*_*k*,*s*_.

As mentioned in the previous section, the deployment of linear search window and update of new vessel direction are both accomplished based on the gradient analysis of specific points. At iteration *k*, in order to get the linear search window, we need to calculate the vessel direction at the extrapolated point *O*_*k*,*s*_. We compare the dominant gradient direction ${\vec{D}}_{g}$ at *O*_*k*,*s*_ and the local vessel direction ${\vec{D}}_{k-1}$ (see Figure 4). The angle difference between these two directions is

(7) $$\begin{array}{c} \Delta \theta ={\hat{\theta }}_{g,O_{k,s}}-\theta_{{\vec{D}}_{k-1}}, \end{array}$$

where ${\hat{\theta }}_{g,O_{k,s}}$ is the angle of the dominant gradient direction at *O*_*k*,*s*_, which can be calculated by (4). According to (5), the angle of the vessel direction at *O*_*k*,*s*_ is

(8) $$\begin{array}{c} \theta_{O_{k,s}}=\{\begin{array}{cc} {\hat{\theta }}_{g,O_{k,s}}-\frac{\pi }{2} & \text{if}\;\;0<\Delta \theta \leq \pi ,\\ {\hat{\theta }}_{g,O_{k,s}}+\frac{\pi }{2} & \text{if}\;\;-\pi <\Delta \theta \leq 0. \end{array} \end{array}$$

Finally, the vessel direction at *O*_*k*,*s*_ is described as a unit vector: ${\vec{D}}_{}{}_{k,s}=(\cos(\theta_{O_{k,s}}),\sin(\theta_{O_{k,s}}))$. The new vessel direction ${\vec{D}}_{}{}_{k}$ is defined as the vessel direction at the new center point *O*_*k*_, which is calculated similarly to the direction at point *O*_*k*,*s*_.

### 3.4. Detection of Branches

The vascular tree in retinal image has complex structures. The linear search window mentioned in Section 3.2 works well when tracking along a vessel without branches. However, it is not suitable when encountering vessel branches. Therefore, we consider using a semicircle search window (see Figure 7(a)) instead of the linear one when branches are predicted.

The choice of different search windows depends on a branch prediction scheme. We have found that a single vessel without branches has two parallel edge lines. Thus, at a given step of the tracking process, we compare the directions at two local vessel edge points (see Figure 4). If the difference between two direction angles exceeds a fixed threshold *T*_angle_, we predict that current blood vessel will bifurcate into new vessel branches. If not, current vessel will be linear.

At iteration *k*, vessel directions at two previous edge points ${\hat{U}}_{k-1}$ and ${\hat{V}}_{k-1}$ are obtained according to (6). Direction angles of the edge points are denoted by $\theta_{{\hat{U}}_{k-1}}$ and $\theta_{{\hat{V}}_{k-1}}$, respectively. The angle difference is calculated as

(9) $$\begin{array}{c} \Delta \theta_{uv}=\left|\theta_{{\hat{U}}_{k-1}}-\theta_{{\hat{V}}_{k-1}}\right|. \end{array}$$

**Figure 5:**
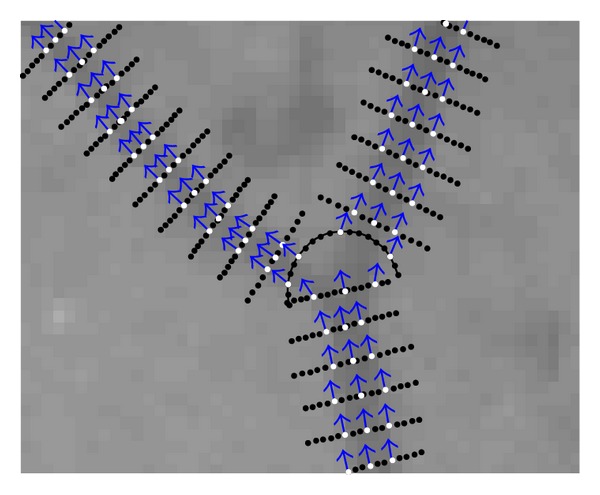
Different search windows during the tracking process. Black points are the candidate edge points and the white ones are the detected vessel edge points and center points. The blue arrows point to the vessel direction at the edge point or center point.

In this study, the threshold *T*_angle_ is chosen as 5°. When Δ*θ*_*uv*_ < *T*_angle_, a linear search window is used. Otherwise, when Δ*θ*_*uv*_ ≥ *T*_angle_, a semicircle search window is used instead of the linear one.  Figure 5 shows the different search windows during the tracking process. However, two vessel edge lines can be sometimes unparalleled due to the noise or eye diseases rather than vessel branches. In these situations, semicircle search window is still used. The proposed method can handle this situation and identify if branches really exist or not.

## 4. Bayesian Method for Vessel Segmentation

### 4.1. Configuration Model

Vessel edge points are detected iteratively by a Bayesian method based on the proposed statistical sampling scheme. In order to choose new edge points among the candidate points on the dynamic search window, we define configuration models by a set of candidate points to describe the possible local vessel's structures. In this study, vessel structures are categorized into three types: normal, bifurcation, and crossing. Normal case is regarded as the situation in which only a single vessel exists in the current search area. The case of bifurcation means that one single vessel bifurcates into two branches. A crossing case is described when one vessel overlaps another. In the proposed method, there are three types of configuration models according to the different vessel structures.

**Figure 6:**
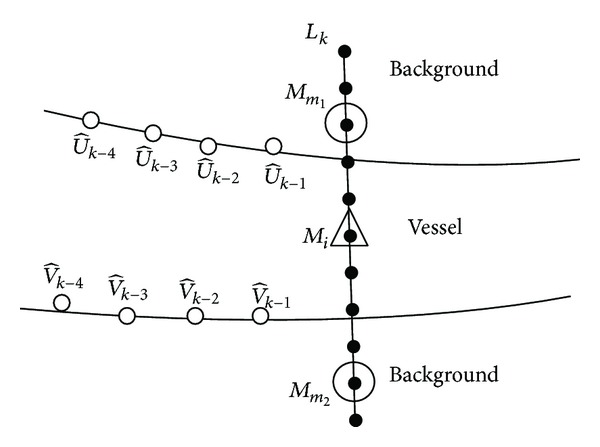
Normal configuration on a linear search window: *M*_*m*_1__ and *M*_*m*_2__ are selected candidate points on linear search window *L*_*k*_. The two selected points give an assumption of local blood vessel and define a normal configuration.

At a given step, if a linear search window is used when local blood vessel is predicted to be linear without branches, we define normal configurations only. As shown in Figure 6, *L*_*k*_ is the linear search window at iteration *k*. We select two candidate points *M*_*m*_1__ and *M*_*m*_2__, which are the *m*_1_^th^ and *m*_2_^th^ points on *L*_*k*_ (1 < *m*_1_ < *m*_2_ < *N*_*L*_*k*__), and assume them as new vessel edge points. The two selected points divide all the candidate points into three parts, two parts belonging to the background and one part belonging to the vessel. Candidate points between the two selected points are assumed to belong to the vessel and others belong to the background. So, the *i*th candidate *M*_*i*_ (*i* ∈ [1, *N*_*L*_*k*__]) is assumed to belong to the blood vessel if *i* ∈ [*m*_1_, *m*_2_] or to the background if *i* ∈ [1, *m*_1_[⋃]*m*_2_, *N*_*L*_*k*__]. Two candidate points selected on the linear search window can be defined as a normal configuration. There are *N*_*L*_*k*__(*N*_*L*_*k*__ − 1)/2 normal configurations at iteration *k*, which is the number of 2 combinations from the set of *N*_*L*_*k*__ candidates on *L*_*k*_.

**Figure 7:**
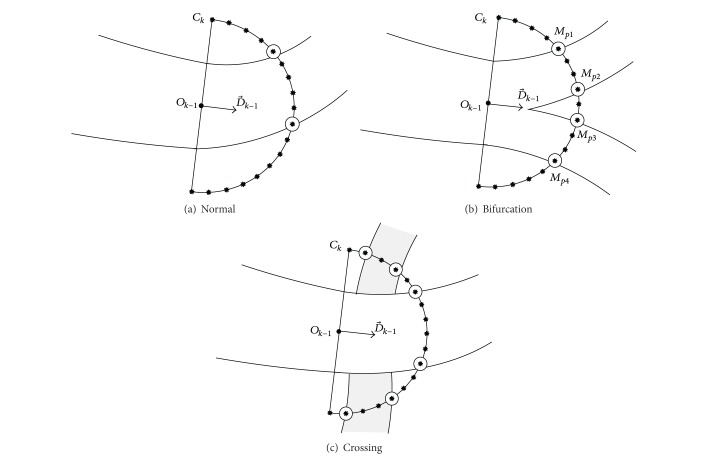
Semicircle search window and three types of configurations: *C*_*k*_ is the semicircle search window at iteration *k*, and points marked by small circles are selected to define different types of configurations.

Otherwise, when vessel branches are predicted, a semicircle search window is used. Three types of configurations (normal, bifurcation, and crossing) are defined. In this situation, the normal configuration is defined to describe the possible local linear blood vessel which is mispredicted as vessel branches by the branch prediction scheme. The bifurcation and crossing configurations are used to describe the possible new vessel branches. At iteration *k*, if a semicircle search window *C*_*k*_ is used, three types of configurations are illustrated in Figure 7. A normal configuration is defined by two selected candidate points on *C*_*k*_, similarly to the definition on the linear search window. For a bifurcation configuration, four candidate points are selected to describe the edge points of two possible branches. Six points are needed for a crossing configuration. Two of the six ones are assumed to be the new edge points of the same vessel, while the other four points are considered as the edge points of another vessel which is over or under the current one.

### 4.2. Probability of Configurations

The configuration models are defined by a set of candidate edge points on the dynamic search window. At a given step, the proposed method attempts to find a configuration, which best matches current vessel structure among all the possible configurations. The best configuration at a given step is obtained by a maximum *a posteriori* estimation. Local vessel structure and new vessel edge points are then detected based on this configuration.

At a given step, by computing the position of each of the *N* candidate edge points on the search window (line segment or semicircle) and assigning the grey level value of the nearest pixel to that point, we obtain the observed discrete intensity profile *Y* = {*y*_*i*_,  *i* = 1,…, *N*}. The posteriori distribution of a configuration *χ* is described as *P*(*χ* | *Y*). According to Bayes' rule,

(10) $$\begin{array}{c} P\left(\chi \;|\;Y\right)=\frac{P\left(Y\;|\;\chi \right)P\left(\chi \right)}{P\left(Y\right)}, \end{array}$$

where *P*(*Y* | *χ*) is the conditional probability of *Y* for a given configuration *χ* and *P*(*χ*) is the *a priori* probability of *χ*. When *N* candidate points are selected, the observed intensity profile *Y* is fixed and has no relationship with the configuration. So, *P*(*Y*) does not depend on the configuration and will be disregarded. Based on the Maximum *a posteriori* (MAP) criterion, the best configuration is obtained as

(11) $$\begin{array}{c} \hat{\chi }=\underset{\chi }{\text{arg max}}\left\{P\left(\chi \;|\;Y\right)\right\}=\underset{\chi }{\text{arg max}}\left\{P\left(Y\;|\;\chi \right)P\left(\chi \right)\right\}. \end{array}$$

#### 4.2.1. Configuration on Linear Search Window

In order to explain (11) in detail, we first discuss the normal configurations on the linear search window. As shown in Figure 6, *L*_*k*_ is the linear search window at iteration *k*. The observed discrete intensity profile on *L*_*k*_ is *Y*_*L*_*k*__ = {*y*_*i*_,  *i* = 1,…, *N*_*L*_*k*__}. A normal configuration, which is defined by two selected candidate points *M*_*m*_1__ and *M*_*m*_2__, is also shown in this figure. Assuming that the discrete grey levels on *L*_*k*_ are independent, the likelihood function of this normal configuration is computed as

(12) $$\begin{array}{c} P\left(Y_{L_{k}}\;|\;\chi \right)=\overset{N_{L_{k}}}{\underset{i=1}{\prod }}P\left(y_{i}\;|\;\chi \right), \end{array}$$

where *P* (*y*_*i*_ | *χ*) is a conditional probability model which is defined to describe the variability of the *i*th candidate point on the search window belonging either to the background or to the blood vessel. As shown in Figure 6, all candidate points on *L*_*k*_ are divided by *M*_*m*_1__ and *M*_*m*_2__ into three parts, which belong to the background, vessel, and background, respectively, so

(13) $$\begin{array}{c} P\left(Y_{L_{k}}\;|\;\chi \right)=\overset{m_{1}-1}{\underset{i=1}{\prod }}P\left(y_{i}\;|\;b\right)\overset{m_{2}}{\underset{m_{1}}{\prod }}P\left(y_{i}\;|\;v\right)\overset{N_{L_{k}}}{\underset{m_{2}+1}{\prod }}P\left(y_{i}\;|\;b\right), \end{array}$$

where *b* and *v* denote background and vessel, respectively.

In Bayesian framework, we assume that *X*_*L*_*k*__ = {*x*_*i*_,  *i* = 1,2,…, *N*_*L*_*k*__} is the true intensity profile associated with the *N*_*L*_*k*__ candidate points on *L*_*k*_. In this study, we assume that the retinal image is only affected by additive noise *ξ*, so

(14) $$\begin{array}{c} Y_{L_{k}}=X_{L_{k}}+\xi . \end{array}$$

**Figure 8:**
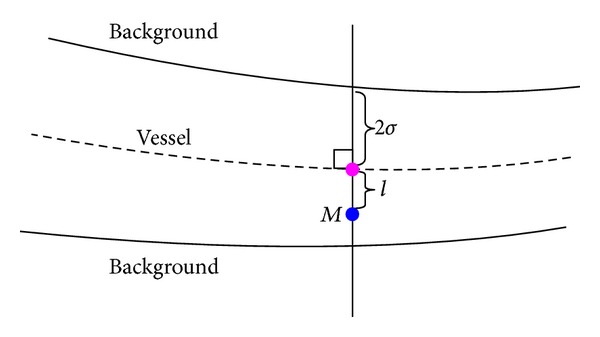
Illustration of (15). The dotted line is the vessel's center line. The pink point is local vessel center point, while the blue one is point *M*.

**Figure 9:**
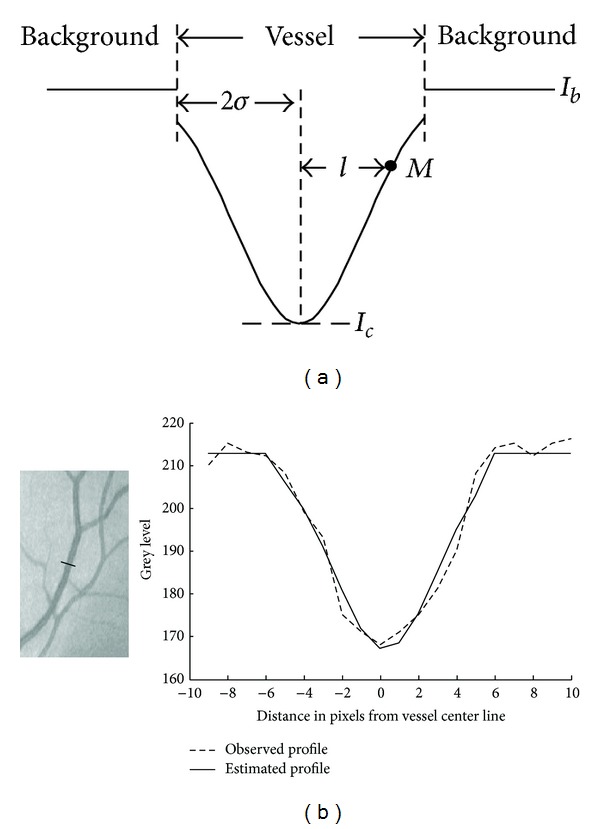
Vessel's sectional intensity profile. (a) Gaussian intensity model. (b) The observed and estimated profiles of the vessel cross-section marked by a black line on the retinal image.

During the tracking process, the local background is assumed to have a constant intensity. Vessel's sectional intensity profile can be approximated by a Gaussian curve [8, 40]. The grey level of a point *M* in local vessel cross-section as shown in Figure 8 is computed as

(15) $$\begin{array}{c} G\left(M\right)=\{\begin{array}{cc} \left(I_{c}-I_{b}\right)\exp\left(-\frac{l^{2}}{2\sigma^{2}}\right)+I_{b} & \text{if}\;\;M\in \text{vessel}\\ I_{b} & \text{if}\;\;M\in \text{background}, \end{array} \end{array}$$

where *I*_*c*_ is the grey level of local vessel's center *I*_*b*_ is the grey level of local background, *l* is the distance between point *M* and the straight line which passes through vessel's center along local vessel direction, and *σ* defines the spread of the intensity profile and is set to the value of half of local vessel's radius. A selected vessel cross-section and its observed and estimated intensity profiles are show in Figure 9. For the normal configuration shown in Figure 6, the true grey level of the *i*th candidate point *M*_*i*_ on *L*_*k*_, is estimated as

(16) xi={xi(v)=(Ic,k−Ib,k)exp⁡(−li22σχ2)+Ib,k          if  i∈[m1,m2]xi(b)=Ib if i∈[1,m1)⋃(m2,NLk],

where *I*_*c*,*k*_ is the grey level of local vessel center and *I*_*b*,*k*_ is the mean grey level of local background areas. *l*_*i*_ is the distance between *M*_*i*_ and the straight line which passes through the middle point of [*M*_*m*_1__, *M*_*m*_2__] and is perpendicular to $\vec{M_{m_{1}}M_{m_{2}}}$. The spread parameter *σ*_*χ*_ = (1/2) | *M*_*m*_1__ *M*_*m*_2__|.

**Figure 10:**
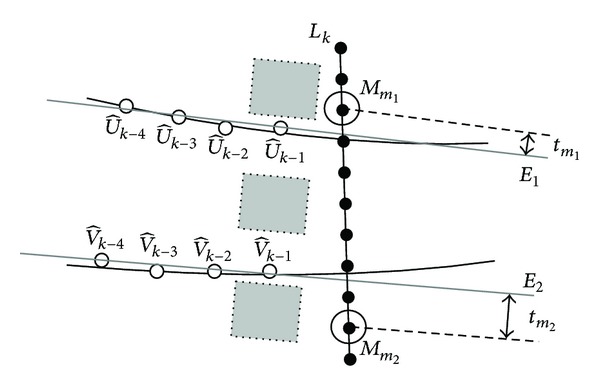
Estimation of vessel edge lines and local statistic parameters.

At iteration *k*, the local intensity parameters *I*_*c*,*k*_ and *I*_*b*,*k*_ are calculated on three moving regions: one inside and two others outside the local blood vessel as shown in Figure 10. These three regions are selected by the algorithm in local research area based on local vessel edge points ${\hat{U}}_{k-1}$ and ${\hat{V}}_{k-1}$. The size of these three regions is adaptive to local vessel diameter. *I*_*c*,*k*_ is the grey level of local vessel center point *O*_*k*−1_. *I*_*b*,*k*_ is the mean grey level of two regions outside local blood vessel.

The observed grey level profile on *L*_*k*_ can be expressed as

(17) yi=xi+ξ={xi(v)+ξvif  i∈[m1,m2]xi(b)+ξbif  i∈[1,m1)⋃(m2,NLk],

where *ξ*_*v*_ and *ξ*_*b*_ are the Gaussian noise in local blood vessel and background, respectively:

(18) $$\begin{array}{c} \xi_{v}\sim N\left(0,\sigma_{v}^{2}\right),\\ \xi_{b}\sim N\left(0,\sigma_{b}^{2}\right). \end{array}$$

The statistical parameters *σ*_*v*_ and *σ*_*b*_ are also computed by three moving regions as shown in Figure 10. *σ*_*v*_ is estimated as the standard deviation of grey levels in the vessel region and *σ*_*b*_ is the standard deviation of grey levels in two background regions. Then, conditional probability model of the proposed normal configuration is

(19) $$\begin{array}{c} P\left(y_{i}\;|\;v\right)=\frac{1}{\sqrt{2\pi }\sigma_{v}}\exp\left(-\frac{{\left(y_{i}-x_{i}\left(v\right)\right)}^{2}}{2\sigma_{v}^{2}}\right),\\ P\left(y_{i}\;|\;b\right)=\frac{1}{\sqrt{2\pi }\sigma_{b}}\exp\left(-\frac{{\left(y_{i}-x_{i}\left(b\right)\right)}^{2}}{2\sigma_{b}^{2}}\right), \end{array}$$

and the likelihood function can be calculated by (13).

In our method, the *a priori* probability is based on the Gibbs formulation [42]:

(20) $$\begin{array}{c} P\left(\chi \right)=\frac{1}{Z}\exp\left(-\lambda U\left(\chi \right)\right), \end{array}$$

where *Z* is a normalization parameter and *λ* is a regularization term. In our study, *Z* was fixed to 1. As *Z* was set to be a constant, it has no influence on the computation of the maximum of (11). After many experiments, *λ* was set to 0.01. *U*(*χ*) is the energy function of a given configuration. Gibbs formulation aims at linking a configuration with an energy function to penalize high energetic configurations.

At a given step, when a linear search window is used, the local blood vessel is assumed to be linear. The local vessel edges can be estimated as two straight lines and new edge points are supposed to be aligned on the local vessel edge lines. At iteration *k* (*k* < 5), the vessel edge lines *E*_1_ and *E*_2_ are estimated as two straight lines along the local direction ${\vec{D}}_{k-1}$ and passing through two edge points ${\hat{U}}_{k-1}$ and ${\hat{V}}_{k-1}$, respectively. When *k* ≥ 5, *E*_1_ and *E*_2_ are defined as the least square straight lines obtained by four pairs of edge points detected in the previous iterations (see Figure 10). Considering the proposed normal configuration of Figure 6, we compute the distance between *M*_*m*_1__ and *E*_1_ and the distance between *M*_*m*_2__ and *E*_2_, which are denoted by *t*_*m*_1__ and *t*_*m*_2__, respectively (see Figure 10). The energy function can be defined as *U*(*χ*) = *t*_*m*_1__^2^ + *t*_*m*_2__^2^ [42]. The *a priori* probability has the expression:

(21) $$\begin{array}{c} P\left(\chi \right)=\frac{1}{Z}\exp\left(-\lambda \left(t_{m_{1}}^{2}+t_{m_{2}}^{2}\right)\right). \end{array}$$

At iteration *k*, the likelihood functions and the *a priori* probabilities of all the *C*_*N*_*L*_*k*___^2^ normal configurations on *L*_*k*_ are computed similarly to the proposed normal configuration of Figure 6. The best configuration at iteration *k* is obtained by (11). The two candidate points used to define the best configuration are regarded as new vessel edge points.

#### 4.2.2. Configurations on Semicircle Search Window

In the other situation, when a semicircle search window is used at a given step, three types of configurations are defined. As shown in Figure 7, *C*_*k*_ is the semicircle search window at iteration *k*. The observed discrete intensity profile on *C*_*k*_ is denoted by *Y*_*C*_*k*__ = {*y*_*i*_,  *i* = 1,…, *N*_*C*_*k*__}. The probabilities of all the configurations on *C*_*k*_ are computed.

For normal configurations on *C*_*k*_, the likelihood function is computed similarly using (13). An example of bifurcation configuration is shown in Figure 7(b). It has four selected points which are the *p*_1_^th^, *p*_2_^th^, *p*_3_^th^, and *p*_4_^th^ candidate points on *C*_*k*_. Its likelihood function can be computed as

(22) P(YCk ∣ χ)=∏i=1p1−1P(yi ∣ b)∏p1p2P(yi ∣ v)∏p2+1p3−1P(yi ∣ b)∏p3p4P(yi ∣ v)∏p4+1NCkP(yi ∣ b).

The sectional intensity distribution of the two assumed vessel branches is also approximated by the Gaussian shaped curve. The true discrete intensity profile on *C*_*k*_ is obtained similarly to (16) according to the given bifurcation configuration. The conditional probability models *P* (*y*_*i*_ | *b*) and *P* (*y*_*i*_ | *v*) in (22) are calculated similarly to (19). In addition, the likelihood function of a crossing configuration is obtained based on its six selected points.

The *a priori* model, which is defined by the previous detected vessel edge points (see (21)), has no influence on bifurcation or crossing configurations. Therefore, the *a priori* probability of a configuration on the semicircle search window, no matter normal, bifurcation, or crossing, is disregarded. According to (11), the best configuration on *C*_*k*_ is obtained as

(23) $$\begin{array}{c} \hat{\chi }=\underset{\chi }{\text{arg max}}\left\{P\left(Y_{C_{k}}\;|\;\chi \right)\right\}. \end{array}$$

If $\hat{\chi }$ is a normal configuration, the local blood vessel is considered to be linear and two candidate points used to define $\hat{\chi }$ are regarded as new vessel edge points at iteration *k*. So, the proposed method solves the problem that local linear blood vessel is mispredicted to bifurcate into vessel branches. If $\hat{\chi }$ is a bifurcation configuration, two vessel branches are detected, and the four selected candidate points are considered as the initial edge points of two branches. If $\hat{\chi }$ is a crossing configuration, current vessel encounters another one. The new detected blood vessel is regarded as two vessel branches starting from the crossing. The third and the fourth selected points of the six ones are regarded as new edge points of current blood vessel. The other four points are considered as the initial edge points of two branches starting from the crossing.

## 5. Experiments and Discussion

The performance of our method was evaluated on three publicly available databases: STARE [6], DRIVE [7], and REVIEW [30, 43]. There are three initial parameters in our algorithm: the distance between grid lines in the selection of seed points, the look-ahead distance *s* (see (2)), and the length of the initial linear window *L*_0_. In our experiments, the distance between grid lines is fixed to 30 pixels, *s* is set to 3 pixels, and *L*_0_ is set to 10 pixels after many trials. These parameter values can give the best results, including computational time, when testing on the three databases.

The STARE database contains 20 retinal images, which are captured by the TopCon TRV-50 fundus camera at a 35° field of view (FOV). 10 of the images are from healthy ocular fundus and the other 10 are from unhealthy ones. All the images are segmented manually by two independent specialists. The proposed method is tested with the segmentation of the first observer as ground truth.

The DRIVE database contains 40 retinal images which are captured by the Canon CR5 camera at 45° FOV. The 40 images were divided into a training set and a test set, each of which contains 20 images. They had been manually segmented by three observers trained by an ophthalmologist. The images in the training set were segmented once, while images in the test set were segmented twice, resulting in sets A and B. Our method is tested on the test set using the segmentations of set A as ground truth.

The REVIEW database consists of four image sets which include 16 images with 193 vessel segments demonstrating a variety of pathologies and vessel types. The high resolution image set (HRIS) represents different sever grades of diabetic retinopathy. The vascular disease image set (VDIS) contains a range of normal and diseased retina, including diabetic and arteriosclerotic retinopathies. The central light reflex image set (CLRIS) represents early atherosclerotic changes with an exaggerated vascular light reflex. The images of the kick point image set (KPIS) are taken from clean, large vessel segments. These four image sets contain 5066 manually marked profiles assessed by three independent experts. The performance of an algorithm can be compared with manual measurement with accuracy up to 0.25 of a pixel.

### 5.1. Segmentation Performance

We used STARE and DRIVE databases to evaluate the segmentation performance of the proposed method. In practice, our algorithm is developed in Matlab (version 7.6.0.324) environment. Some functions are programmed using C language in order to save computation time. The average running time of our tracking method on one image is 9.6 min on STARE and 6.3 min on DRIVE. Our runtimes are higher than the ones obtained using nontracking vessel segmentation. For example, the processing time of Mendonça's method [1] is 3 min for a STARE image, and 2.5 min for a DRIVE image. Mendonça and Campilho [1] used a pixel-based global approach and also implemented their algorithm using Matlab. In order to assess the proposed method and compare it with the other state-of-art methods, we use three widely known performance measures: the detection accuracy, sensitivity, and specificity. The accuracy is defined as the ratio of the total number of correctly classified pixels to the number of pixels in the FOV (field of view). The sensitivity is defined as the ratio of the number of correctly classified vessel pixels to the number of total vessel pixels in the ground truth. The specificity is defined as the ratio of the number of correctly classified nonvessel pixels to the number of total nonvessel pixels inside FOV in the ground truth.

**Figure 11:**
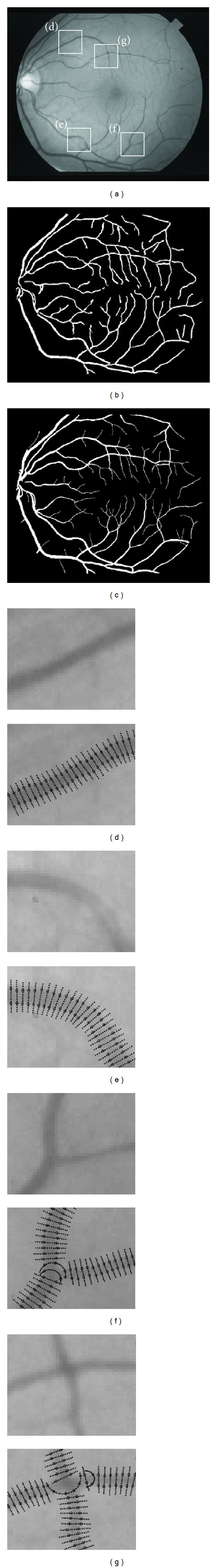
Tests on different vessel structures. (a) Retinal image (*im0077*) from the STARE database. Four selected regions show different vessel structures such as line, curve, bifurcation, and crossing. (b) Segmentation result by the proposed method. (c) Ground truth. (d)–(g) Subimages obtained from (a) and the related dynamic search window and detected vessel edge points.

First, we discuss the segmentation performance of the proposed method on 20 retinal images from the STARE database. For example, we discuss the test result of our method on a retinal image (*im0077*) from the STARE database as shown in Figure 11(a). The segmentation result of the proposed method and the ground truth are shown in Figures 11(b) and 11(c), respectively. In order to obtain the final detected blood vessels, we first obtain vessel edge lines by linking the detected vessel edge points. Then, the pixels between two vessel edge lines are considered to belong to the blood vessel. By comparing with the ground truth, we can see that the proposed method is able to detect most of the blood vessels. The detection sensitivity, specificity, and accuracy of the proposed method on *im0077* are 0.8020, 0.9600, and 0.9426, respectively. Besides, we selected four regions on *im0077* as shown in Figure 11(a), which present four types of vessel structures: line, curve, bifurcation, and crossing. The enlarged regions are shown in Figures 11(d)–11(g), respectively. In the second row of Figures 11(d)–11(g), we can see that the linear search window is used when tracking for linear blood vessels, while the semicircle search window is used to detect vessel branches in the bifurcation or crossing structures. The candidate points on each search window are marked by block dots, while detected vessel edge points are marked by small circles.

Three methods, Hoover's [6], Soares' [13] and Mendonça's [1] methods, are used for comparison purposes on the STARE database. Hoover et al. [6] proposed a filter-based method and made their segmentation results on the STARE database public on their website. Soares' [13] method is based on the 2D Gabor wavelet transform, and its test results on the STARE database are presented on the public website (http://retinal.sourceforge.net/). Mendonça and Campilho [1] used morphological operators for vessel segmentation.  Table 1 lists the segmentation performance of different methods on the STARE database. As mentioned in the beginning of this section, segmentation results of the first observer are used for the ground truth. In Table 1, the manual detection results of the second observer are considered as a reference used for comparison. The performance measures of Hoover's [6] and Soares' [13] methods are calculated using the segmented images from their public websites, respectively. The performance of Mendonça's method is obtained from the original paper [1]. The experimental results show that our method has a higher sensitivity (0.7248) than the other three methods. The specificity and accuracy of the proposed method are 0.9666 and 0.9412, respectively, which are both higher than those of the second observer and Hoover's method [6].

**Figure 12:**
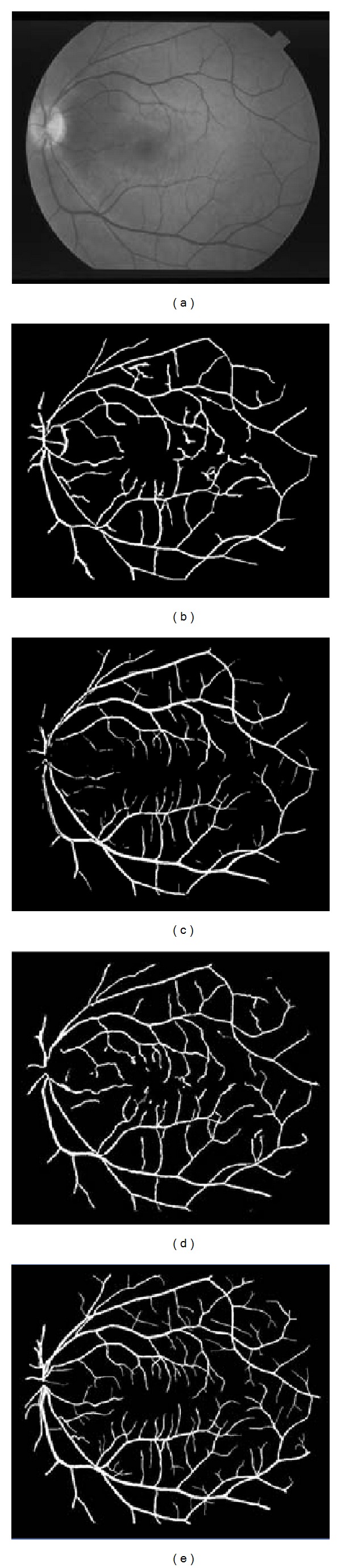
Results on a normal retinal image from the STARE database: (a) retinal image (*im0255*); (b) Hoover et al. [6]; (c) Soares et al. [13]; (d) the proposed method; (e) ground truth.

**Figure 13:**
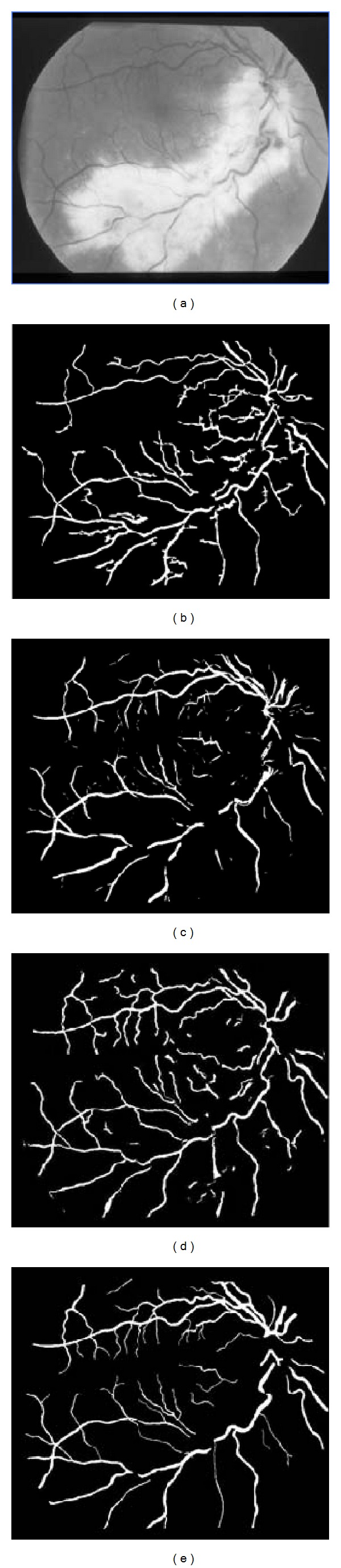
Results on an abnormal retinal image from the STARE database: (a) retinal image (*im0044*); (b) Hoover et al. [6]; (c) Soares et al. [13]; (d) the proposed method; (e) ground truth.

As mentioned above, the STARE database includes 10 normal and 10 abnormal retinal images. In Table 2, we show the performance measures of different methods in the two cases on STARE database. In normal cases, the proposed method outperforms Hoover's method [6] and has a similar performance to Mendonça's method [1]. In abnormal cases, the proposed method is better than the other three methods. The sensitivity of the proposed method is up to 0.7034, which is much higher than that of the others. To make the comparison fairer, we show the segmentation results of different methods on two retinal images from the STARE database, one normal *im0255* and one abnormal *im0044* (see Figures 12 and 13). The ground truth image is the segmentation result of the first observer. Result images of Hoover's [6] and Soares' [13] methods are downloaded directly from their public website (http://retinal.sourceforge.net/). In the normal case, as shown in Figure 12, the proposed method detected more details than the others, especially in the area near the fovea. In the abnormal case, as shown in Figure 13, our method detected most of the vascular tree, while Hoover's method lost some thin blood vessels and the main blood vessels in Soares' result are discontinued.

Secondly, we discuss the segmentation performance on DRIVE database. Several popular vessel segmentation methods are considered when testing on the DRIVE database for comparison purposes as shown in Table 3. The performance measures of the compared methods are obtained from the website of DRIVE database (http://www.isi.uu.nl/Research/Databases/DRIVE/). We can see that the proposed method has higher specificity and accuracy than those of the others. The sensitivity is a bit inferior to some state-of-the-art methods. Because our method seems to be effective in abnormal cases as shown in Table 2, most of the images are normal in DRIVE database.

**Figure 14:**
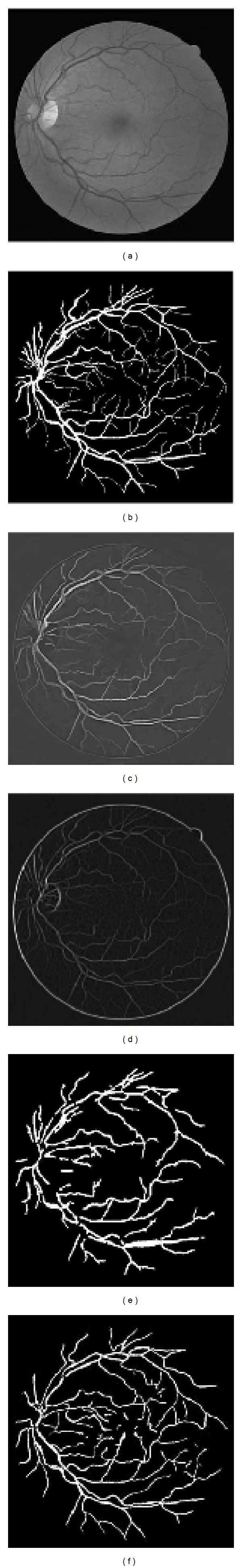
Results on a retinal image from DRIVE database: (a) retinal image (*01_test*); (b) ground truth; (c) Martínez-Pérez et al. [31]; (d) Chaudhuri et al. [8]; (e) Jiang and Mojon [9]; (f) the proposed method.

Figure 14 shows the segmentation results of four different methods on the retinal image *01_test* from the DRIVE database. As shown in Figure 14(a), *01_test* is a normal retinal image without retinopathy. The ground truth as shown in Figure 14(b) is manual segmentation result. Figures 14(c) and 14(d) show the original classification results of the Martnez-Pérez's [31] and Chaudhuri's [8] algorithms, respectively. For these two methods, the final segmentation results are obtained from the original results by thresholding at a certain value. Jiang and Mojon [9] use the adaptive local thresholding and the segmentation result is shown in Figure 14(e). There are disconnected main blood vessels and missed thin blood vessels in Jiang's results. The segmentation result of the proposed method is shown in Figure 14(f).

### 5.2. Width Measurement Performance

**Figure 15:**
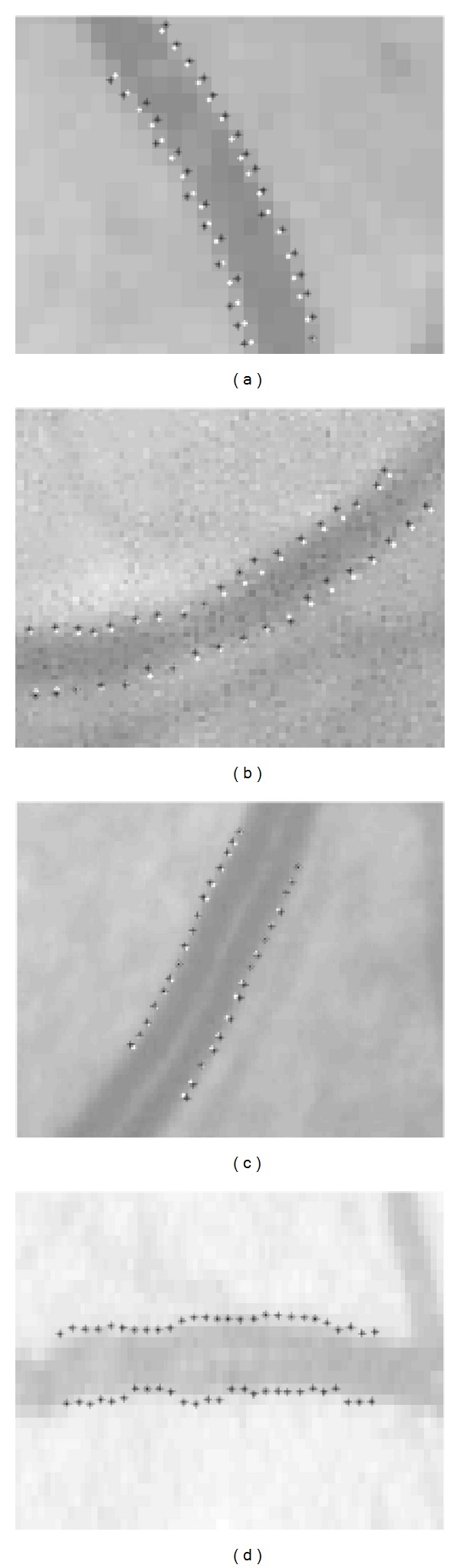
Test results of the proposed method on the REVIEW database. Images (a)–(d) show the vessel segments obtained from HRIS, VDIS, CLRIS, and KPIS, respectively.

To assess the diameter measurement performance, we have used the four datasets (HRIS, VDIS, CLRIS, and KPIS) of the REVIEW database. In REVIEW database, the profiles marked by the observers provide the locations of vessel edge points for the vessel segments in HRIS, VDIS, and CLRIS. Local vessel parameters are calculated based on these marked vessel edge points. If a pair of local marked vessel edge points are denoted by (*x*_1_, *y*_1_) and (*x*_2_, *y*_2_), the local vessel center point is ((*x*_1_ + *x*_2_)/2, (*y*_1_ + *y*_2_)/2), diameter is $\sqrt{{\left(x_{1}-x_{2}\right)}^{2}+{\left(y_{1}-y_{2}\right)}^{2}}$, and direction is (*π*/2) + arctan((*y*_2_ − *y*_1_)/(*x*_2_ − *x*_1_)). For the dataset KPIS, the manually marked profiles supply the vessel center point, diameter, and direction directly, without giving the vessel edge points. When testing our method on the images of REVIEW database, the first profile of each selected vessel segment is used for the initialization. The tracking process is stopped when the search area exceeds the selected vessel segment.  Figure 15 shows examples of vessel segments obtained from the four datasets, respectively. The edge points detected by the proposed method are marked by black stars, while the ground truth points given by the database are marked by white dots. Because the database does not give the observed edge points in KPIS, there are no ground truth points in Figure 15(d).

**Figure 16:**
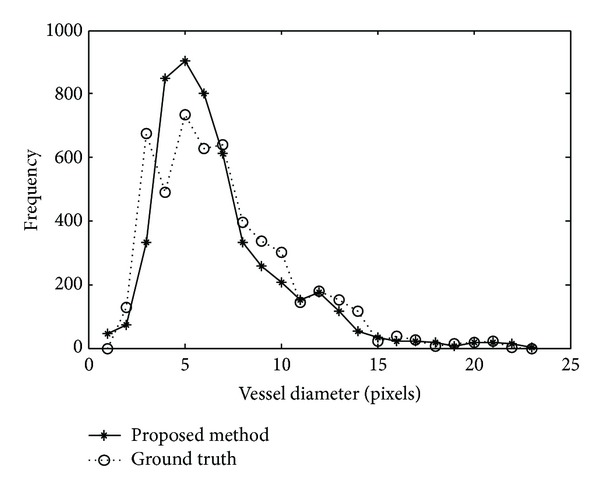
Distribution of vessel diameters on the REVIEW database.

It is known that the actual vessel diameters in the REVIEW database are obtained by observers. In order to compare the actual diameters with the diameters detected by the proposed method, we analyze the distribution of the vessel diameters. For this purpose, the distribution of the diameters is obtained by two steps: rounding the values of vessel diameters to integers and, then, computing the frequency of the diameters. The distribution curves of the actual and the detected vessel diameters on the REVIEW database are shown in Figure 16. The actual distribution curve is marked by stars, while the detected distribution is marked by circles. From the distribution curves, we can see that most of the blood vessels have diameters between 3 and 10 pixels. The largest blood vessel does not exceed 23 pixels in width. The proposed method has a high accuracy in vessel width measurement.

We have also compared the performance of the proposed method with the half height full width (HHFW) [38], Gregson's [37], 1D Gaussian [39], 2D Gaussian [40], and Al-Diri's [30] methods by presenting the success rate and mean width. When an algorithm fails to detect the vessel width at a given point (e.g., does not converge), the current width measurement is considered meaningless. The success rate of an algorithm is defined as the ratio between the number of meaningful measurements and the number of total measurements.  Table 4 shows the performance of the different methods by presenting the success rate and the mean width. For each dataset, the success rate is shown in the first column and the mean width is shown in the second column. The results of three observers are obtained from the database. For each dataset, the mean result of the three observers is used as ground truth. The results of the compared methods are obtained from the original paper of Al-Diri. We can see from the success rates that the HHFW, 1D Gaussian, 2D Gaussian, and Al-Diri's methods failed to detect some vessel segments on datasets HRIS, VDIS, and CLRIS. In particular, the success rate of the HHFW is down to 0 on the CLRIS. For Gregson's and the proposed methods, the success rate is 100% on all of the four datasets. However, the proposed method has higher accuracy in the mean width than Gregson's method. In general, the proposed method outperforms all the other algorithms, especially on the CLRIS dataset. On the HRIS and VDIS datasets, our method is slightly inferior to Al-Diri's method, which, however, did not use all its measurement results.

## 6. Conclusions

In this paper, we have introduced an automatic tracking method for vessel detection in retinal images. The tracking algorithm is able to segment the main vascular tree and obtain local vessel parameters such as vessel edge points, direction, and the vessel width along the retinal vessels. The proposed method starts from a number of seed points selected all over the image. During the tracking process, a dynamic search window is used to get the information of local grey levels' statistics. Different types of configurations are defined to combine the intensity distribution and the geometrical structure of local blood vessel. Vessel's edge points are detected by a Bayesian method with the MAP criterion. Experiments on three publicly available databases show that the proposed method detects blood vessels effectively and outperforms some classical vessel detection methods. We compared our raw results with the other techniques. Some postprocessing will be done later in order to improve the detection accuracy. Besides, the improvements of vessel sectional intensity model and the reconstruction method of the detected vascular tree are both necessary work in the future.

## Figures and Tables

**Table 1:** Evaluation results of different segmentation methods on STARE database.

Method	Sensitivity	Specificity	Accuracy
The 2nd observer	0.8949	0.9390	0.9354
Hoover et al. [6]	0.6751	0.9567	0.9267
Soares et al. [13]	0.7165	0.9748	0.9480
Mendonça and Campilho [1]	0.6996	0.9730	0.9440
Proposed	**0.7248**	**0.9666**	**0.9412**

**Table 2:** Evaluation results on normal and abnormal retinal images from STARE database.

Method	Sensitivity	Specificity	Accuracy
Normal cases			
The 2nd observer	0.9646	0.9236	0.9283
Hoover et al. [6]	0.6766	0.9662	0.9324
Soares et al. [13]	0.7554	0.9812	0.9542
Mendonça and Campilho [1]	0.7258	0.9791	0.9492
Proposed	**0.7463**	**0.9664**	**0.9405**
Abnormal cases			
The 2nd observer	0.8252	0.9544	0.9425
Hoover et al.[6]	0.6736	0.9472	0.9211
Soares et al. [13]	0.6869	0.9682	0.9416
Mendonça and Campilho [1]	0.6733	0.9669	0.9388
Proposed	**0.7034**	**0.9668**	**0.9420**

**Table 3:** Evaluation results of different segmentation methods on DRIVE database.

Method	Sensitivity	Specificity	Accuracy
The 2nd observer	0.7761	0.9725	0.9473
Al-Diri et al. [30]	0.7282	0.9551	0.9258
Jiang and Mojon [9]	0.6478	0.9625	0.9212
Martínez-Pérez et al. [31]	0.7086	0.9496	0.9181
Chaudhuri et al. [8]	0.2716	0.9794	0.8773
Proposed	**0.6522**	**0.9710**	**0.9267**

**Table 4:** Performance of different methods on REVIEW database.

	HRIS		VDIS		CLRIS		KPIS	
Method	%	Mean	%	Mean	%	Mean	%	Mean
Observer	100	4.35	100	8.85	100	13.8	100	7.52
Gregson et al. [37]	100	7.64	100	10.07	100	12.8	100	7.29
HHFW [38]	88.3	4.97	78.4	7.94	0	—	96.3	6.47
1D-G [39]	99.6	3.81	99.9	5.78	98.6	6.3	100	4.95
2D-G [40]	98.9	4.18	77.2	6.59	26.7	7.0	100	5.87
Al-Diri et al. [30]	99.7	4.63	99.6	8.8	93.0	15.7	100	6.56
Proposed	**100**	**4.81 **	**100**	**7.85**	**100**	**13.39**	**100**	**6.71**

## References

[1] Mendonça AM, Campilho A (2006). Segmentation of retinal blood vessels by combining the detection of centerlines and morphological reconstruction. *IEEE Transactions on Medical Imaging*.

[2] Fang B, Hsu W, Lee ML On the detection of retinal vessels in fundus images. http://hdl.handle.net/1721.1/3675.

[3] Kirbas C, Quek F (2004). A review of vessel extraction techniques and algorithms. *ACM Computing Surveys*.

[4] Lesage D, Angelini ED, Bloch I, Funka-Lea G (2009). A review of 3D vessel lumen segmentation techniques: models, features and extraction schemes. *Medical Image Analysis*.

[5] Fraz MM, Remagnino P, Hoppe A (2012). Blood vessel segmentation methodologies in retinal images—a survey. *Computer Methods and Programs in Biomedicine*.

[6] Hoover A, Kouznetsova V, Goldbaum M (2000). Locating blood vessels in retinal images by piecewise threshold probing of a matched filter response. *IEEE Transactions on Medical Imaging*.

[7] Staal J, Abràmoff MD, Niemeijer M, Viergever MA, van Ginneken B (2004). Ridge-based vessel segmentation in color images of the retina. *IEEE Transactions on Medical Imaging*.

[8] Chaudhuri S, Chatterjee S, Katz N, Nelson M, Goldbaum M (1989). Detection of blood vessels in retinal images using two-dimensional matched filters. *IEEE Transactions on Medical Imaging*.

[9] Jiang X, Mojon D (2003). Adaptive local thresholding by verification-based multithreshold probing with application to vessel detection in retinal images. *IEEE Transactions on Pattern Analysis and Machine Intelligence*.

[10] Sofka M, Stewart CV (2006). Retinal vessel centerline extraction using multiscale matched filters, confidence and edge measures. *IEEE Transactions on Medical Imaging*.

[11] Zana F, Klein JC (2001). Segmentation of vessel-like patterns using mathematical morphology and curvature evaluation. *IEEE Transactions on Image Processing*.

[12] Niemeijer M, Staal J, van Ginneken B, Loog M, Abràmoff MD Comparative study of retinal vessel segmentation methods on a new publicly available database.

[13] Soares JVB, Leandro JJG, Cesar RM, Jelinek HF, Cree MJ (2006). Retinal vessel segmentation using the 2-D Gabor wavelet and supervised classification. *IEEE Transactions on Medical Imaging*.

[14] Ricci E, Perfetti R (2007). Retinal blood vessel segmentation using line operators and support vector classification. *IEEE Transactions on Medical Imaging*.

[15] Kozerke S, Botnar R, Oyre S, Scheidegger MB, Pedersen EM, Boesiger P (1999). Automatic vessel segmentation using active contours in cine phase contrast flow measurements. *Journal of Magnetic Resonance Imaging*.

[16] Gooya A, Liao H, Matsumiya K, Masamune K, Masutani Y, Dohi T (2008). A variational method for geometric regularization of vascular segmentation in medical images. *IEEE Transactions on Image Processing*.

[17] Vasilevskiy A, Siddiqi K (2002). Flux maximizing geometric flows. *IEEE Transactions on Pattern Analysis and Machine Intelligence*.

[18] Law MWK, Chung ACS (2009). Efficient implementation for spherical flux computation and its application to vascular segmentation. *IEEE Transactions on Image Processing*.

[19] Kayikcioglu T, Mitra S A new method for estimating dimensions and
3-d reconstruction of coronary arterial trees from biplane angiograms.

[20] Sato Y, Araki T, Hanayama M, Naito H, Tamura S (1998). A viewpoint determination system for stenosis diagnosis and quantification in coronary angiographie image acquisition. *IEEE Transactions on Medical Imaging*.

[21] Wang L, Bhalerao A, Wilson R (2007). Analysis of retinal vasculature using a multiresolution hermite model. *IEEE Transactions on Medical Imaging*.

[22] Tolias YA, Panas SM (1998). A fuzzy vessel tracking algorithm for retinal images based on fuzzy clustering. *IEEE Transactions on Medical Imaging*.

[23] Can AH, Shen H, Turner JN, Tanenbaum HL, Roysam B (1999). Rapid automated tracing and feature extraction from retinal fundus images using direct exploratory algorithms. *IEEE Transactions on Information Technology in Biomedicine*.

[24] Zou P, Chan P, Rockett P (2009). A model-based consecutive scanline tracking method for extracting vascular networks from 2-D digital subtraction angiograms. *IEEE Transactions on Medical Imaging*.

[25] Chutatape O, Zheng L, Krishnan S Retinal blood vessel detection and tracking by matched gaussian and kalman filters.

[26] Grisan E, Pesce A, Giani A, Foracchia M, Ruggeri A A new tracking system for the robust extraction of retinal vessel structure.

[27] Ng J, Clay ST, Barman SA (2010). Maximum likelihood estimation of vessel parameters from scale space analysis. *Image and Vision Computing*.

[28] Yedidya T, Hartley R Tracking of blood vessels in retinal images using kalman filter.

[29] Vlachos M, Dermatas E (2010). Multi-scale retinal vessel segmentation using line tracking. *Computerized Medical Imaging and Graphics*.

[30] Al-Diri B, Hunter A, Steel D (2009). An active contour model for segmenting and measuring retinal vessels. *IEEE Transactions on Medical Imaging*.

[31] Martínez-Pérez ME, Hughes AD, Stanton AV, Thom SA, Bharath AA, Parker KH Retinal blood vessel segmentation by means of scale-space analysis and region growing.

[32] Zhou S, Chen W, Zhang Z, Yang J Automatic segmentation of coronary angiograms based on probabilistic tracking.

[33] Florin C, Paragios N, Williams J Particle filters, a quasi-monte carlo solution for segmentation of coronaries.

[34] Al-Kofahi KA, Lasek S, Szarowski DH (2002). Rapid automated three-dimensional tracing of neurons from confocal image stacks. *IEEE Transactions on Information Technology in Biomedicine*.

[35] Adel M, Moussaoui A, Rasigni M, Bourennane S, Hamami L (2010). Statistical-based tracking technique for linear structures detection: application to vessel segmentation in medical images. *IEEE Signal Processing Letters*.

[36] Yin Y, Adel M, Bourennane S (2012). Retinal vessel segmentation using a probabilistic tracking method. *Pattern Recognition*.

[37] Gregson PH, Shen Z, Scott RC, Kozousek V (1995). Automated grading of venous beading. *Computers and Biomedical Research*.

[38] Brinchmann-Hansen O, Heier H (1986). Theoretical relations between light streak characteristics and optical properties of retinal vessels. *Acta Ophthalmologica*.

[39] Zhou L, Rzeszotarski MS, Singerman LJ, Chokreff JM (1994). Detection and quantification of retinopathy using digital angiograms. *IEEE Transactions on Medical Imaging*.

[40] Lowell J, Hunter A, Steel D, Basu A, Ryder R, Kennedy RL (2004). Measurement of retinal vessel widths from fundus images based on 2-D modeling. *IEEE Transactions on Medical Imaging*.

[41] Rao AR, Jain RC (1992). Computerized flow field analysis: oriented texture fields. *IEEE Transactions on Pattern Analysis and Machine Intelligence*.

[42] Ibañez MV, Simó A (1999). Bayesian detection of the fovea in eye fundus angiographies. *Pattern Recognition Letters*.

[43] Al-Diri B, Hunter A, Steel D, Habib M, Hudaib T, Berry S Review—a reference data set for retinal vessel profiles.

